# Targeting Tumor Necrosis Factor-α with Adalimumab: Effects on Endothelial Activation and Monocyte Adhesion

**DOI:** 10.1371/journal.pone.0160145

**Published:** 2016-07-28

**Authors:** Raghav Oberoi, Jutta Schuett, Harald Schuett, Ann-Kathrin Koch, Maren Luchtefeld, Karsten Grote, Bernhard Schieffer

**Affiliations:** Department of Cardiology and Angiology, Philipps-University Marburg, Marburg, Germany; Monash University, AUSTRALIA

## Abstract

**Objective:**

It is well known that atherosclerotic inflammatory vascular disease is critically driven by oxidized lipids and cytokines. In this regard, tumor necrosis factor (TNF)-α is known as a crucial mediator of early pro-atherosclerotic events. Epidemiologic data suggest that blockade of TNF-α has beneficial effects on vascular outcomes in patients with rheumatoid arthritis, however, detailed mechanistic studies are still lacking. This study aims to elucidate effects of TNF-α blockade by adalimumab–which is approved for several inflammatory disorders–on endothelial activation and monocyte adhesion under pro-atherosclerotic conditions.

**Methods and Results:**

Phorbol myristate acetate (PMA) differentiated THP-1 macrophages were stimulated with oxidized low density lipoprotein and subsequent analysis of this conditioned media (oxLDL CM) revealed a strong release of TNF-α. The TNF-α rich supernatant led to activation of human umbilical vein endothelial cells (HUVEC) as shown by enhanced expression of major adhesion molecules, such as vascular cell adhesion molecule-1 (VCAM-1), intercellular adhesion molecule-1 (ICAM-1) and E-selectin which was suppressed by the TNF-α inhibitor adalimumab. Accordingly, adalimumab effectively prevented THP-1 monocyte adhesion to endothelial cells under static as well as under flow conditions. Furthermore, adalimumab suppressed endothelial leakage as shown by Evan's blue diffusion across a confluent endothelial monolayer. Of note, after intraperitoneal injection we detected abundant deposition of fluorophore-labelled adalimumab in atherosclerotic plaques of hypercholesterolemic mice.

**Conclusion:**

Our results show that adalimumab prevents major inflammatory effects of TNF-α on endothelial activation, endothelial monocyte adhesion, endothelial leakage and therefore extends the therapeutic options of adalimumab to limit vascular inflammation.

## Introduction

Atherosclerosis is a chronic inflammatory disease characterized by accumulation of lipids and fibrous elements in the large arteries and is known as the major contributor to the growing burden of cardiovascular disease (CVD) [1]. Elevated levels of blood low density lipoproteins (LDL) and endothelial dysfunction are considered as pre-disposition for atherogenesis. Hyperlipidemia can lead to accumulation of LDL molecules within the intima of arteries where it undergoes modifications such as glycation or oxidation [2]. Vascular inflammation is driven by many different cytokines predominantly derived from activated macrophages.

TNF-α is such a cytokine which has been implicated to play a key role in the pathogenesis of atherosclerosis [3] and other chronic inflammatory diseases like asthma, chronic obstructive pulmonary disease, rheumatoid arthritis and inflammatory bowel disease [4–7]. The greatest advancement in good prognosis of rheumatoid arthritis over the last decade has been made due to the identification of the pivotal role of TNF-α in its pathogenesis. Of note, although many other cytokines are involved in the progression of the disease but TNF-α has been shown to play a major role and TNF-α blockers are already approved for therapy for quite a while [8]. With regard to molecular and cellular processes, rheumatoid arthritis and atherosclerosis have much in common. Inflamed synovium and atherosclerotic plaque are similar in a number of aspects. Both scenarios are characterized by the presence of large amount of inflammatory cytokines and monocytes/macrophages [9]. In this regard, TNF-α is established as a potent inducer of endothelial and epithelial cell adhesion molecule expression such as vascular cell adhesion molecule-1 (VCAM-1) [10], intercellular adhesion molecule-1 (ICAM-1) [11] and E-selectin [12]. Studies using up-to-date medical techniques such as flow mediated dilation and laser Doppler perfusion imaging has demonstrated improved endothelial dysfunction in rheumatoid arthritis patients upon TNF-α blocker therapy [13].

Adalimumab (HUMIRA, Abbott) is one of the leading therapies for the treatment of rheumatoid arthritis. It is a humanized monoclonal antibody that binds to TNF-α and blocks its interaction with the TNF receptor [14]. It neutralizes both soluble as well as transmembrane TNF-α. Adalimumab has demonstrated a good prognosis and improvement of physical function in rheumatoid arthritis [8,15]. The important role of TNF-α for atherosclerotic plaque development in experimental models is well documented, different TNF-α-deficient mice models consistently showed reduced plaque burden [16–18]. However, effects of pharmacological inhibition of TNF-α on fundamental pro-atherosclerotic processes are still poorly investigated. Therefore, we investigated the potential of the TNF-α blocker adalimumab on endothelial activation with subsequent monocyte adhesion and endothelial leakage under pro-atherosclerotic conditions.

## Material and Methods

### Mice

All experiments were approved by the governmental animal ethics committee at Philipps University Marburg and performed according to the guidelines of the Federation of European Animal Science Associations. Male C57BL/6J Ldlr^‒/‒^ (B6.129S7-Ldlrtm1Her/J) mice from our own breeding mice were maintained in the Central Animal Facility at Philipps-University Marburg. Mice at the age of 8 weeks were fed a high-fat, high-cholesterol diet (D12108, Research Diets, New Brunswick, NJ) for 6 weeks. At the end of the experiment, mice were analgized and euthanized with a mixture of ketamine (120 mg/kg) and xylazine (12 mg/kg) followed by blood withdrawal from the left ventricle.

### Recombinant proteins, lipoproteins and antibodies

Recombinant human TNF-α and human macrophage colony-stimulating factor (M-CSF) was purchased from Miltenyi Biotec (Bergisch Gladbach, Germany). Human oxidized low density lipoprotein (oxLDL), Dil(1,1´-dioctadecyl-3,3,3`,3`-tetramethylindocarbo-cyanine perchlorate)-labelled oxLDL and native LDL (nLDL) was from Kalen Biomedical LLC (Montgomery Village, MD). Antibodies for Western blot against ICAM-1, E-selectin were obtained from R&D Systems (Minneapolis, MN) and VCAM-1, Glyceraldehyde-3-phosphate dehydrogenase (GAPDH) from Santa Cruz (Dallas, TX). An antibody for immunofluorescence against VE-cadherin was obtained from Santa Cruz. CD11b and CD11c antibodies labelled with allophycocyanin (APC) and PerCP/Cy5.5 respectively for flow cytometry were obtained from BioLegend (San Diego, SA). Appropriate secondary antibodies were from Jackson ImmunoResearch (West Grove, PA) Adalimumab (HUMIRA) was obtained from Abbott GmbH (Wiesbaden, Germany). Human IgG control antibody, 4',6-diamidino-2-phenylindole (DAPI) and lipopolysaccharide (LPS) were obtained from Sigma-Aldrich (Munich, Germany).

### Cells

Human monocytic cell line (THP-1) was obtained from Cell Lines Service (Eppelheim, Germany). Cell were cultured in RPMI-1640 (Roswell Park Memorial Institute) media containing 2 mM L-glutamine, 10 mM HEPES, 1 mM sodium pyruvate, 4.5 g/L glucose and 1.5 g/L sodium bicarbonate. In addition, media was supplemented with 10% FCS, 1% penicillin/streptomycin (Sigma-Aldrich) and 50 μM β-mercaptoethanol (Sigma-Aldrich). Cells up to passage 20 were used for the experiments. For differentiation, THP-1 monocytes were plated at a density of 0.5x10^6^ cells per milliliter in complete media supplemented with 50 ng/mL of phorbol 12-myristate 13-acetate (PMA, Sigma-Aldrich) in 6 well plates (Corning, Tewksbury, MA) and incubated for 48 hours. After incubation, cells referred to as THP-1 macrophages were adherent. Cells were washed with complete media to ensure maximal removal of PMA. After washing, cells were used for experiment.

Peripheral blood mononuclear cells (PBMCs) were isolated from buffy coats using ficoll (Biochrom GmbH, Berlin) density gradient centrifugation. 2x10^7^ PBMCs were seeded in 6 well plates (Corning) and allowed to adhere on the surface of the plates for 45 minutes in media (RPMI-Glutmax supplemented with 10% FCS and 1% penicillin/streptomycin). Non-adhered cells were removed with warm PBS, media containing recombinant human M-CSF (50 ng/mL) was added and cells were cultured for 8 days and subsequently used for experiments. Medium was changed on day 4 and 6.

Human endothelial cells (human umbilical vein endothelial cells, HUVECs) were obtained from Promocell (Heidelberg, Germany). Cells were cultured in endothelial growth medium (EGM; Promocell) supplemented with 2% fetal calf serum (FCS, PAN Biotech, Aidenbach, Germany), growth factors (epidermal growth factor, vascular endothelial growth factor 165, basic fibroblast growth factor, insulin-like growth factor) and heparin in 75 cm^2^ cell culture flasks (Sarstedt, Nürnbrecht, Germany). Cells were kept in endothelial cell basal medium (EBM, Promocell) supplemented with 1% FCS without growth factors before and during experiments. Cells between passage 2 and 4 were used for the experiments.

### Tissue preparation

Preparation of mouse tissues was performed as described previously [19]. Briefly, after withdrawal of blood from the left ventricle, heart and aorta were removed after perfusion with PBS. The thoraco-abdominal aorta was fixed in 3.7% formalin for the measurement of atherosclerotic burden. Liver and spleen were embedded and snap-frozen in Tissue Tek OCT (Sakura Finetek, Staufen, Germany) for immunohistochemistry.

### Fluorescence labeling of adalimumab

In order to analyze the vascular distribution of adalimumab, we labelled the substance using the DyLight™ 549 Microscale Antibody Labeling Kit according to the manufacturer’s protocol (Thermo Scientific, Rockford, IL). Briefly, one vial of DyLight™ 549 Reagent was dissolved in 1 mg adalimumab solution (in PBS; PAA Laboratories, Colbe, Germany) and purified with a resin spin column to remove excess fluor. Hypercholesterolemic Ldlr^‒/‒^ mice were injected with fluorophore-labelled adalimumab (8.0 mg/kg in 200 μl PBS i.p.) for two consecutive days and sacrificed 12 hours after last injection. Vehicle injection (200 μl PBS) was used as control. Subsequently, the thoraco-abdominal aorta was removed, opened longitudinally, pinned on a black silicone-covered dish under a stereomicroscope (Stemi DV4, Carl Zeiss Microimaging, Jena, Germany). Subsequently, it was photographed under an epi-fluorescence microscope using appropriate filter sets from Axio Vert.A1 microscope with an AxioCam MRm camera (Carl Zeiss, Jena, Germany).

### Histochemistry and immunohistochemistry

Atherosclerotic burden of the thoraco-abdominal aorta were visualized by en face oil red O-staining as described previously [19]. After epi-fluorescence microscopy the pinned thoraco-abdominal aorta was formalin-fixed and stained with propylene glycol-dissolved oil red O (2 hours at room temperature). The pinned aorta was photographed under PBS immersion using a stand-equipped camera (EOS 600D, Canon, Tokyo, Japan).

Serial cryostat sections of liver and spleen tissue (8 μm, HM-500 O cryostat, Microm, Walldorf, Germany) were produced and stored at -20°C. For staining, sections were air-dried, fixed in ice-cold acetone and stained with an anti-human IgG Fc antibody (Rockland, Gilbertsville, PA) or corresponding IgG control (Santa Cruz) and with a horseradish peroxidase-conjugated secondary antibody (Dianova, Hamburg, Germany), followed by incubation with DAB substrate (Vector Laboratories, Burlingame, CA) and counter stained with hematoxylin (Sigma-Aldrich).

### Flow cytometry

Differentiation of THP-1 macrophages was analyzed using flow cytometry. Briefly, THP-1 monocytes differentiated with PMA or vehicle control dimethyl sulfoxide (DMSO, Sigma-Aldrich) were stained for 20 min on ice for the THP-1 macrophage maturation marker CD11b and CD11c antibodies labelled with APC and PerCP/Cy5.5, respectively. Appropriate IgG-APC was used as isotype control. Cells were acquired on FACS LSR II flow cytometer (BD Biosciences, San Jose, CA) and analyzed using flowjo (Tree Star Inc., Ashland, OR).

### Preparation of conditioned media

THP-1 macrophages were stimulated with 25 and 50 μg/mL oxLDL for 48 hours. After stimulation, supernatant was collected, pre-cleared by centrifugation and passed through 0.2 μm filters and kept at 4°C. This supernatant is referred to as oxLDL conditioned media (oxLDL CM), conditioned media from unstimulated THP-1 macrophages were used as control (control CM).

### Real-time polymerase chain reaction (PCR)

0.5x10^5^ HUVECs were plated in 12-well plates (Corning) in 1 ml EGM per well and grown till confluency. Cells were starved in EBM with 1% FCS for 6 hours. After starvation, cells were stimulated with conditioned media with or without adalimumab or IgG (both 1 μg/mL) for the mentioned time. Total RNA was isolated using RNeasy mini kit (Qiagen, Hilden, Germany) and reverse-transcribed with SuperScript reverse transcriptase kit (Applied Biosystems, Darmstadt, Germany). Real Time PCR was performed in duplicates in a total volume of 20 μL using Power SYBR® green PCR master mixture (Applied Biosystems) on a Step OnePlus Real-Time PCR system (Applied Biosystems) in 96-well PCR plates (Applied Biosystems). Real-time PCR was done with an initial denaturation step at 95°C for 10 min followed by 40 PCR cycles consisting of 95°C for 15 s, 60°C for 1 min, and 72°C for 1 min, and SYBR green fluorescence emission were monitored after each cycle. For normalization, expression of β-actin was determined in duplicates. Fold change with respect to control was calculated using the 2^–ΔΔCT^ method. PCR primers were obtained from TIB MOLBIOL (Berlin, Germany). Primer sequences are as follows: VCAM-1 forward: 5’-TGT TTG CAG CTT CTC AAG CTT TT-3’, VCAM-1 reverse: 5’-GAT GTG GTC CCC TCA TT CGT-3’, ICAM-1 forward: 5’-AGC TTC GTG TCC TGT ATG GC-3’, ICAM-1 reverse: 5’- TTT CTG GCC ACG TCC AGT TT-3’, E-selectin forward: 5’-GCC TGC AAT GTG GTT GAG TG-3’, E-selectin reverse: 5’-ACG AAC CCA TTG GCT GGA TT-3’, P-selectin forward: 5’-TGA TAA TGG GTG GGA CGC TC-3’, P-selectin reverse: 5’-TTT ATG GAA ACC TTA AGG ACT CGG G-3’, CD36 forward: 5’-GGC CAT ACA CCT ACA GGG AAC-3’, CD36 reverse: 5’-GCA CCT GGG ACC ACT CTA TG-3’, LOX1 forward: 5’-CAG CCC CAT CCA GAA TGG AAA-3’, LOX1 reverse: 5’-TCG GGC TCA TTT AAC TGG GAA-3’, β-actin forward: 5’-CAT GTA CGT TGC TAT CCA GGC-3’, β-actin reverse: 5’-CTC CTT AAT GTC ACG CAC GAT-3’.

### Western blotting

5x10^5^ HUVECs were plated in 60 mm dishes (Corning) in 4 ml EGM per well and grown till confluency. Cells were starved as mentioned before. After starvation, cells were stimulated with conditioned media with or without adalimumab (1 μg/mL) for 6 hours. IgG (1 μg/mL) was used as isotype control. Total protein was extracted with buffer containing 150 mM NaCl, 1% Triton X-100, 0.5% sodium deoxycholate, 0.1% SDS, 50 mM Tris supplemented with protease inhibitor cocktail (Roche, Penzberg, Germany). Total protein content was measured using the BCA kit (Thermo Scientific, Waltham, MA) according to manufactures protocol. 30 μg of total protein was loaded onto 10% denaturing SDS gel and transferred to 0.45 μm polyvinylidene difluoride (PVDF) membrane (Amersham Biosciences, Amersham, UK) for immunoblotting. Membrane was blocked with 5% non-fat dry milk, (Sigma-Aldrich) and probed with primary antibody against proteins of interest followed by horseradish peroxidase (HRP)-labelled secondary antibody. Proteins were detected using chemiluminescence substrate (BioRad Laboratories, Hercules, USA). Results were documented on a Chemo-star imaging system (INTAS, Göttingen, Germany). Signal intensity of chemiluminescence was quantified using Quantity One software (BioRad).

### Enzyme-linked immunosorbent assay (ELISA)

Supernatants from THP-1 macrophages and primary human macrophages after oxLDL stimulation were analyzed for TNF-α by ELISA from R&D Systems according to the manufacturers’ protocols and analyzed on an Infinite M200 pro plate reader (TECAN Instruments, Maennedorf, Switzerland). LPS (100 ng/mL) was used as positive control.

### Protein array

Supernatants from THP-1 macrophages after stimulation with oxLDL and with or without adalimumab (1 μg/mL) were analyzed with commercially available protein array for human cytokines and growth factor related to inflammation according to the manufacturer’s instructions (RayBiotech, Norcross, GA). Briefly, antibody array membranes were blocked for 30 min with blocking buffer followed by incubation with 1 mL of undiluted supernatants overnight at 4°C. After washing, membranes were incubated with detection antibody cocktail overnight at 4°C, followed by incubation with HRP-streptavidin for again overnight at 4°C. Membranes were developed using chemiluminescence detection method and results were documented on a Chemo-star imaging system (INTAS). Signal intensity of chemiluminescence was quantified using Quantity One software (BioRad Laboratories).

### Cell cytotoxicity (alamar blue)

1 x 10^4^ HUVECs were plated per well of 96 well plate (Corning) in EGM. After reaching sub-confluency cells were cultured in different concentration of adalimumab (0.01–10 μg/mL), IgG isotype control (10 μg/mL) and 0.1% Triton-X (Sigma-Aldrich) as positive control in 5% CO_2_ at 37°C. After 12 hours, 10% Alamar blue (Pierce, Thermo Scientific) was added to each well and kept in incubator for another 6 hours. Absorbance of oxidized alamar blue was measured at 600 nm on Infinite M200 pro plate reader (TECAN Instruments). All measurements were performed in triplicate.

### Foam cell formation

Foam cell formation of THP-1 macrophages was analyzed by their ability to take up oxLDL labelled with the lipophilic tracer Dil. Therefore, PMA-differentiated THP-1 macrophages were incubated with Dil-labelled oxLDL or Dil-labelled nLDL (each 10 μg/mL) for 4 hours in RPMI-1640 supplemented with 1% FCS. Nuclei were visualized using DAPI. Foam cell formation was evaluated by FACS (LSR II flow cytometer) and by a DM550B fluorescence microscope with a DFC300FX camera (Leica Microsystems, Wetzlar, Germany).

### Adhesion assay

#### Adhesion under static conditions

3x10^4^ HUVECs were plated per well of a 48 well plate (Corning) in EGM and grown to complete confluence. Cells were starved in EBM supplemented with 1% FCS for 6 hours. Following starvation, cells were incubated with conditioned media with or without adalimumab (1 μg/mL) for 4 hours. TNF-α (10 ng/mL) was used as positive control and IgG (1 μg/mL) was used as isotype control. In parallel THP-1 monocytes were labelled with 5 μM of CellTracker™ green (Invitrogen) according to the manufacturer’s instructions. After stimulation, HUVECs were washed twice with 500 μL EBM and 0.5x10^6^ labelled THP-1 monocytes were added per well of a 48 well plate and incubated for 30 min in 5% CO_2_ at 37°C. After incubation each well was washed three times with 500 μL EBM and 10 high power field (HPF) digital images were taken using Axio Vert.A1 microscope with an AxioCam MRm camera (Carl Zeiss, Jena, Germany). Adhered cells per HPF was counted using ImageJ software (National Institute of Health, USA).

#### Adhesion under flow conditions

Flow based adhesion assay was done as described by Shetty et al. [20]. Briefly, 5x10^5^ HUVECs were plated in μ-Slide I 0.4 flow chambers (ibidi GmbH, Munich, Germany) in EGM and grown to complete confluence. Cells were starved in EBM supplemented with 1% FCS for 6 hours. Following starvation, cells were incubated with conditioned media with or without adalimumab (1 μg/mL) for 4 hours. TNF-α (10 ng/mL) was used as positive control and IgG (1 μg/mL) was used as isotype control. After stimulation, μ-Slide was connected to a 20 cm silicon tubing attached to a luer lock adaptor (B Braun AG, Melsungen, Germany) which was in turn connected to a 50 mL syringe attached to a Perfusor VII pump (B Braun AG). For flow conditions, a flow rate of 0.53 mL/min (which corresponds to laminar flow of 0.5 dyne/cm^2^) was maintained. Endothelial layer was perfused with EBM for 2 min to remove any debris and dead cells. After washing, THP-1 monocytes at a concentration of 1x10^6^ cells/mL were perfused for 5 min at a constant shear stress of 0.5 dyne/cm^2^, followed by perfusion with EBM for 5 min to remove unbound cells. The last 2 min of washing with EBM were recorded by a DM550B fluorescence microscope with a DFC300FX camera (Leica Microsystems) and 20 HPF digital images from this recording were subsequently used for the analysis. Adhered cells per high power field were counted using ImageJ software.

### Migration assay

Transwell inserts (Corning) with 8 μm pores were coated with fibronectin (20 μg/mL, Roche, Germany) for 30 min at 37°C. 700 μL of conditioned media was added to each well of a 24 well plate (Corning) followed by addition of fibronectin coated inserts. To each insert 1x10^5^ THP-1 monocytes were added and cells were allowed to migrate for 12 hours in 5% CO_2_ at 37°C. TNF-α (10 μg/mL) was used as control and IgG (1 μg/mL) was used as isotype control. For staining inserts were washed with phosphate buffered saline (Sigma-Aldrich) followed by fixation with 3.7% formalin solution (Roth) for 10 min. Following fixation inserts were stained with 0.2% crystal violet (Sigma-Aldrich). Cells on the top of the insert were removed using a cotton swab and only cells which migrated across the membrane were analyzed. The membrane of the transwell insert were carefully cut using a scalpel and mounted on a glass slide using mounting medium (VectaMount™ AQ, Vector Laboratories). 10 high power field (HPF) digital images were taken using DMI 3000B microscope with a DFC300FX camera (Leica Microsystems). Cells per HPF were counted using ImageJ software.

### Permeability assay

Permeability of HUVECs cultured on transwell inserts was assayed using Evans blue bound to bovine serum albumin (BSA). Briefly, 2x10^4^ HUVECs were cultured on transwell inserts (Greiner bio-one, Frickenhausen, Germany) with 0.4 μm pore size in EGM. At day 3, EGM in the luminal (upper) chamber was replaced by conditioned media with or without adalimumab or IgG (1 μg/mL) for 4 hours. Following stimulation, inserts were washed with PBS with Ca^++^ and Mg^++^. Afterwards, 300 μL of 0.002% Evans blue bound to 0.1% BSA (Sigma-Aldrich) in PBS with Ca^++^ and Mg^++^ was added to the luminal chamber and 1 mL PBS with Ca^++^ and Mg^++^ was added to the abluminal (lower) chamber. After 3 hours, 100 μL from the abluminal chamber was removed and absorbance was measured at 620 nm using Infinite M200 pro plate reader (TECAN Instruments). TNF-α (10 ng/mL) was used as control.

In parallel, 5x10^4^ HUVECs were cultured on chamber slides (Sarstedt) and treated as mentioned before. Afterwards, cells were fixed in 4% paraformaldehyde for 10 min, blocked with 1% BSA, and stained with an antibody against VE-cadherin at +4°C overnight followed by an appropriated TRITC-labelled secondary antibody. Nuclei were visualized using DAPI. Coverslips were mounted onto glass slides using mounting medium (VectaShield^®^, Vector Laboratories). Images were captured using a DM550B fluorescence microscope with a DFC300FX camera (Leica Microsystems).

### Statistical analysis

All data are represented as mean with standard error of the mean (SEM). Data were compared using the 2-tailed Student *t* test for independent samples or one-way ANOVA followed by Tukey multiple comparison test when more than two groups were compared (GraphPad Prism, version 6.05, GraphPad Software, Inc., USA. *P* value of less than 0.05 was considered statistically significant. Numbers of replicated experiments are indicated in each figure legend. RT-PCR and ELISA measurements were performed in duplicates.

## Results

### OxLDL-dependent release of TNF-α and other inflammatory factors from THP-1 macrophages

**Fig 1:**
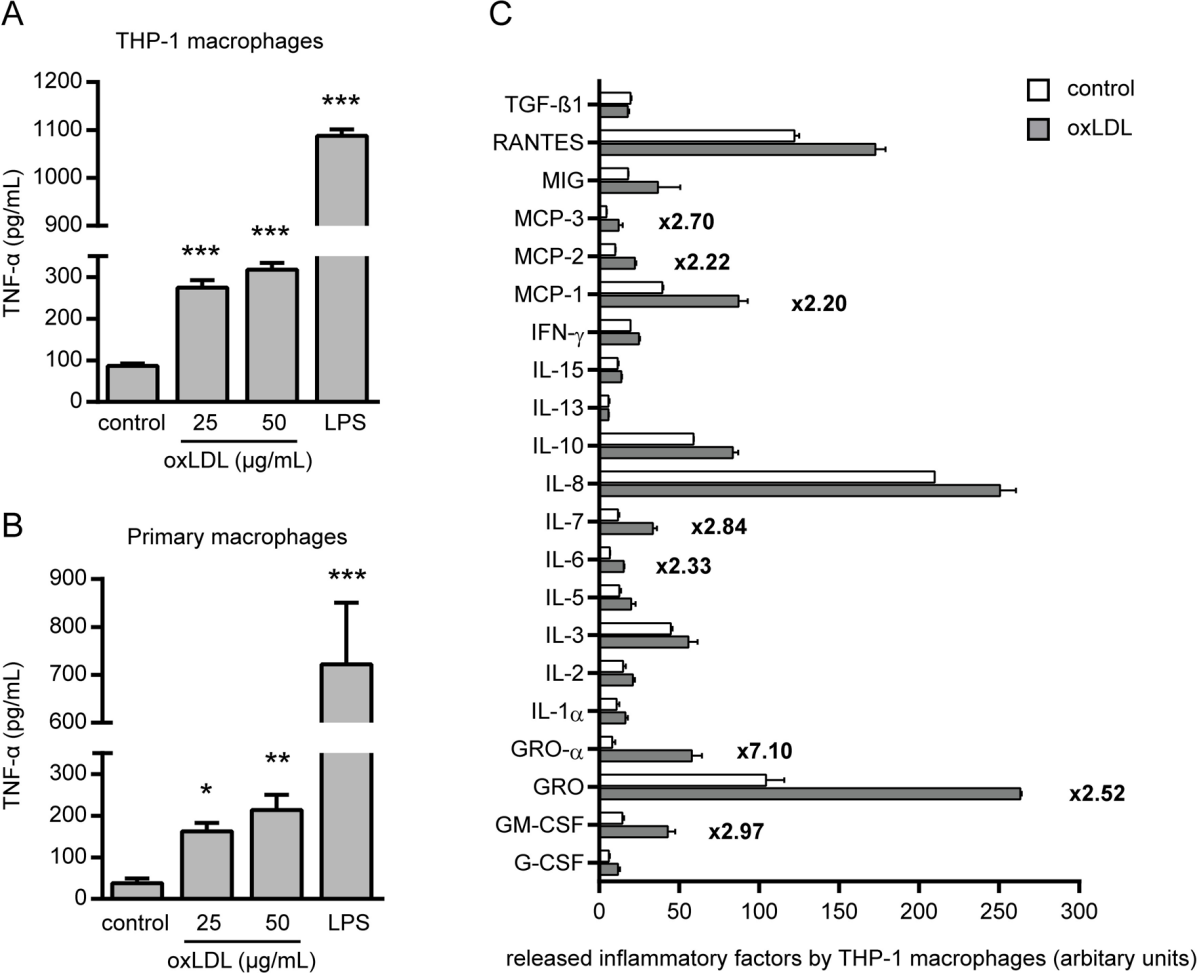
OxLDL-dependent release of TNF-α and other inflammatory factors from THP-1 macrophages. TNF-α release from (A) THP-1 macrophages following 48 hours or (B) primary human macrophages following 24 hours of oxLDL (25 and 50 μg/mL) stimulation was determined by ELISA. LPS (100 ng/mL) was used as positive control. *P<0.05, **P<0.01, ***P<0.001 vs. control, n = 4–6 replicated experiments. (C) Conditioned medium from THP-1 macrophages following 48 hours of oxLDL (25 μg/mL) was analyzed by protein array. Conditioned medium from unstimulated THP-1 macrophages were used as control. Bar graph showing densitometric analysis of array membranes specific for inflammation-related factors. Factors with more than 2-fold increase after stimulation are indicated in the graph.

Deposition of oxLDL in the arterial wall and subsequent vascular invasion of monocytes which differentiate into macrophages and release cytokines such as TNF-α is known as a key event in atherosclerotic plaque development [1,2,21]. To mimic these processes, we differentiated human monocytic THP-1 cells by treatment with PMA for 48 hours into macrophages (S1A Fig). Cells became adhered and up-regulated the THP-1 macrophage maturation markers CD11b and CD11c as confirmed by flow cytometry (S1B Fig) [22] as well as the scavenger receptors CD36 and LOX1 as determined by RT-PCR (S1C Fig). Subsequently, THP-1 macrophages were treated with oxLDL or left untreated for additional 48 hours and conditioned medium was collected (S1A Fig). Characteristics of foam cells due to uptake of oxLDL by these cells were demonstrated by FACS (S1D Fig) and fluorescence microscopy (S1E Fig) using Dil-labelled oxLDL and nLDL preparations. As already reported by others [23], analysis of the conditioned medium (hereinafter referred to as oxLDL CM and control CM) by ELISA revealed an oxLDL-induced TNF-α release from THP-1 macrophages (Fig 1A) that was confirmed in additional control experiments using primary human macrophages (Fig 1B). To explore which other pro-inflammatory factors were oxLDL-dependently released from THP-1 macrophages we took advantage of protein arrays. Additional prominent cytokines and chemokines were identified which were at least 2-fold induced, e.g. GROα, IL-6, MCP-1 (Fig 1C).

### The TNF-α inhibitor adalimumab suppresses endothelial activation by oxLDL-stimulated THP-1 macrophages

**Fig 2:**
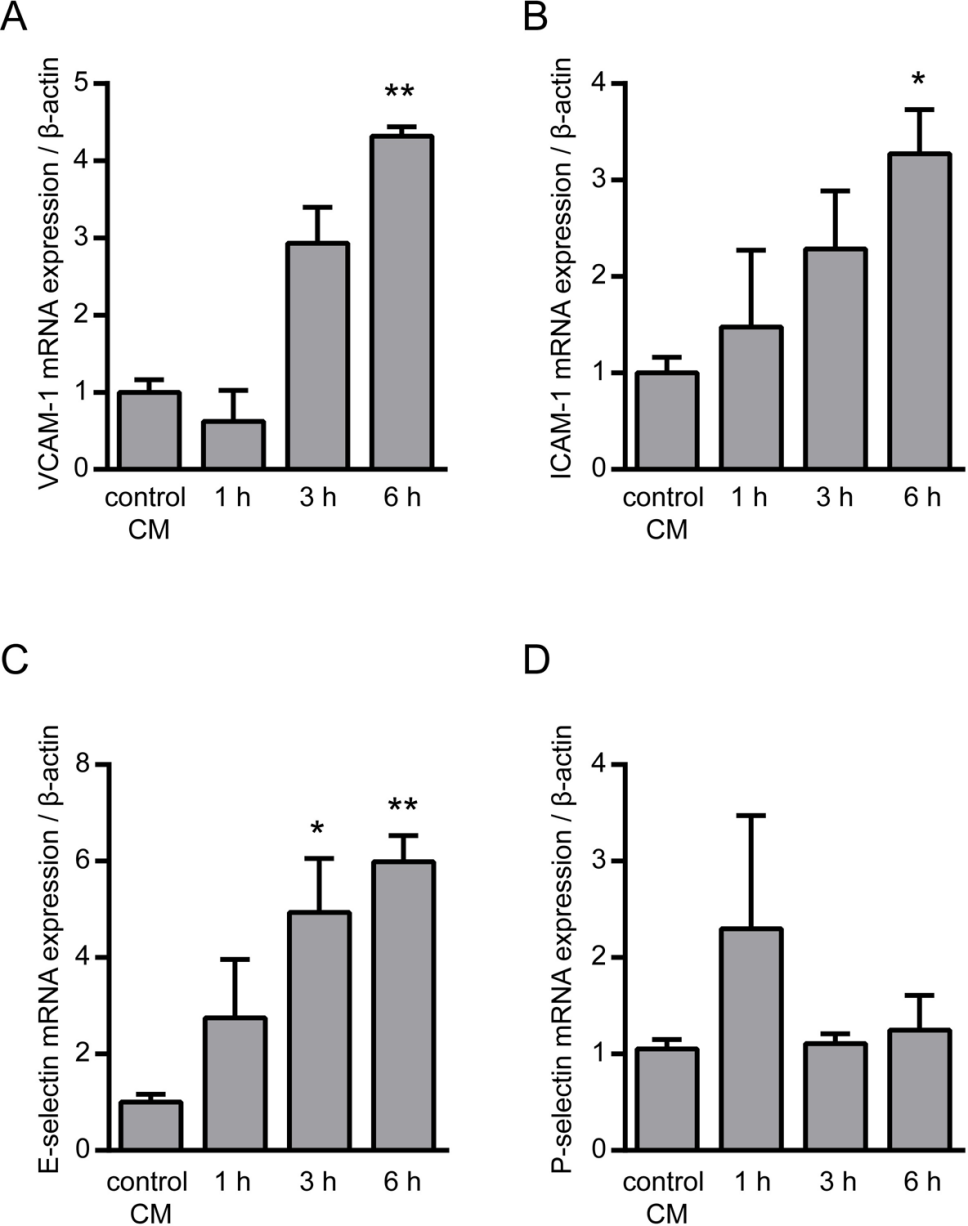
Effect of conditioned media from oxLDL-stimulated THP-1 macrophages on endothelial adhesion molecules. mRNA expression of the adhesion molecules (A) VCAM-1, (B) ICAM-1, (C) E-selectin and (D) P-selectin in HUVECs following stimulation with conditioned media from oxLDL stimulated THP-1 macrophages (oxLDL CM) was determined by real time PCR at the indicated time points. Conditioned medium from unstimulated THP-1 macrophages were used as control (control CM). *P<0.05, **P<0.01, ***P<0.001 vs. control CM, n = 4–5 replicated experiments.

**Fig 3:**
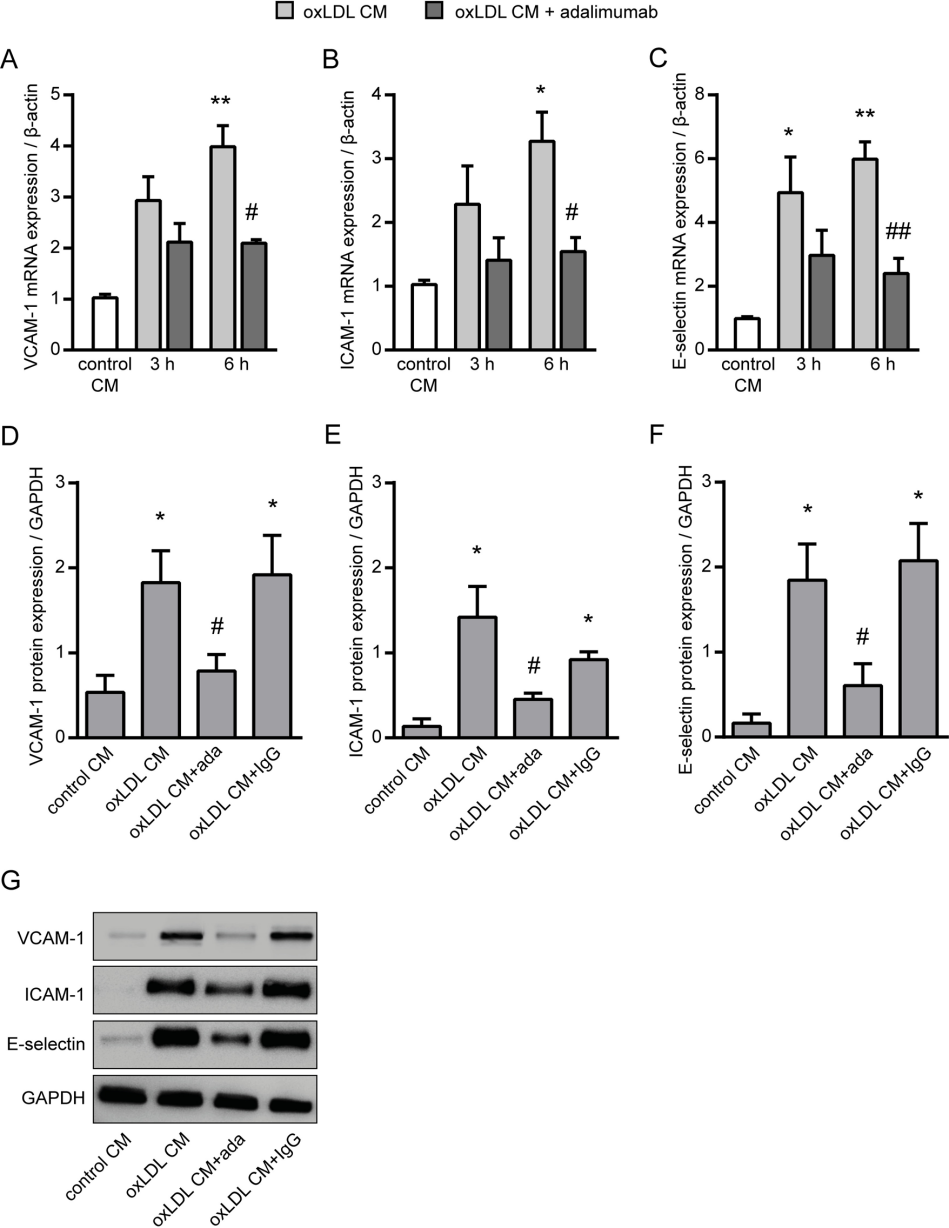
Effect of TNF-α inhibition with adalimumab on endothelial adhesion molecules. mRNA expression of the adhesion molecules (A) VCAM-1, (B) ICAM-1 and (C) E-selectin in HUVECs following 6 hours of incubation with conditioned media from oxLDL-stimulated THP-1 cells (oxLDL CM) with or without adalimumab (ada, 1 μg/mL) was determined by real time PCR. Conditioned medium from unstimulated THP-1 macrophages were used as control (control CM). Protein expression of the adhesion molecules (D) VCAM-1, (E) ICAM-1 and (F) E-selectin in HUVECs following 6 hours of incubation with conditioned media from oxLDL-stimulated THP-1 cells (oxLDL CM) with or without adalimumab was determined by western blot. (G) Representative blots are shown. Conditioned medium from unstimulated THP-1 macrophages (control CM) and IgG isotype (1 μg/mL) were used as control. GAPDH was used as loading control for normalization. *P<0.05, **P<0.01, ***P<0.001 vs. control CM, ^#^P<0.05, ^##^P<0.01 vs. oxLDL CM, n = 6–8 replicated experiments.

Using the before generated oxLDL CM we studied early pro-atherosclerotic processes and a potential intervention with the TNF-α inhibitor adalimumab on these processes. First, we investigated endothelial activation which is such an early event. As expected, oxLDL CM was found to be highly potent to facilitate endothelial activation as shown by enhanced mRNA expression of major adhesion molecules in HUVECs. The mRNA expression of VCAM-1, ICAM-1 and E-selectin was significantly up-regulated 3 to 6 hours after stimulation–a process known to be stimulated by TNF-α [10–12]–whereas mRNA expression of P-selectin was unchanged (Fig 2A–2D). Accordingly, inhibition of TNF-α by addition of adalimumab to the oxLDL CM significantly prevented the up-regulated mRNA expression as well as protein expression of the endothelial adhesion molecules VCAM-1, ICAM-1 and E-selectin in HUVECs after 6 hours (Fig 3A–3G). Of note, treatment of HUVECs with adalimumab for up to 12 hours shows no adverse effect on their cell viability, even with a 10-fold higher concentration of adalimumab than used in this study (S2 Fig).

### The TNF-α inhibitor adalimumab suppresses adhesion of THP-1 monocytes to endothelial cells

**Fig 4:**
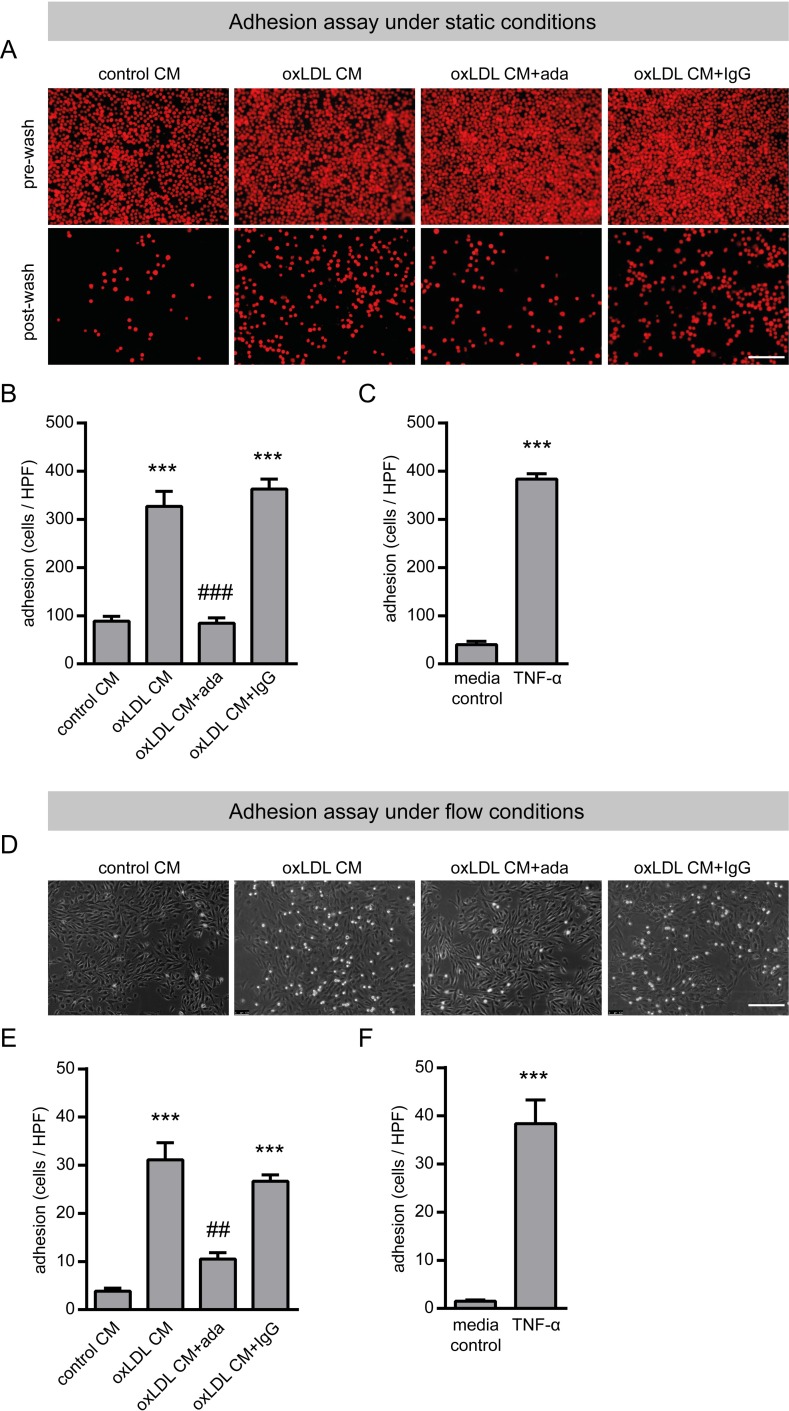
Effect of TNF-α inhibition with adalimumab on adhesion of THP-1 monocytes to endothelial cells. Adhesion assay under static conditions. (A) Fluorescence images depicting CellTracker green-labelled THP-1 monocytes on a HUVECs monolayer after incubation with conditioned media from oxLDL-stimulated THP-1 macrophages (oxLDL CM) with or without adalimumab (ada) for 4 hours followed by the addition of CellTracker green-labelled THP-1 monocytes. Pictures before and after washing with basal medium are shown. (B) Adherent cells per high power field were quantified after washing. (C) HUVECs monolayer was stimulated with TNF-α (10 ng/mL) for 4 hours followed by the addition of CellTracker green-labelled THP-1 monocytes. Adherent cells per high power field were quantified after washing. Adhesion assay under flow conditions. (D) Phase contrast images of THP-1 monocytes on a HUVECs monolayer after incubation with conditioned media from oxLDL-stimulated THP-1 macrophages (oxLDL CM) with or without adalimumab (ada) for 4 hours followed by the addition of THP-1 monocytes with a flow rate of 0.53 mL/min (0.5 dyne/cm^2^). (E) Adherent cells per high power field were quantified after washing. (F) HUVECs monolayer was stimulated with TNF-α (10 ng/mL) for 4 hours followed by the addition of THP-1 monocytes with a flow rate of 0.53 mL/min (0.5 dyne/cm^2^). Adherent cells per high power field were quantified after washing. Conditioned medium from unstimulated THP-1 macrophages (control CM), IgG isotype (1 μg/mL) and medium were used as control. Scale bar = 200 μm. Representative pictures are shown. ***P<0.001 vs. control CM or medium, ^##^P<0.01, ^###^P<0.001 vs. oxLDL CM, n = 3–4 replicated experiments.

**Fig 5:**
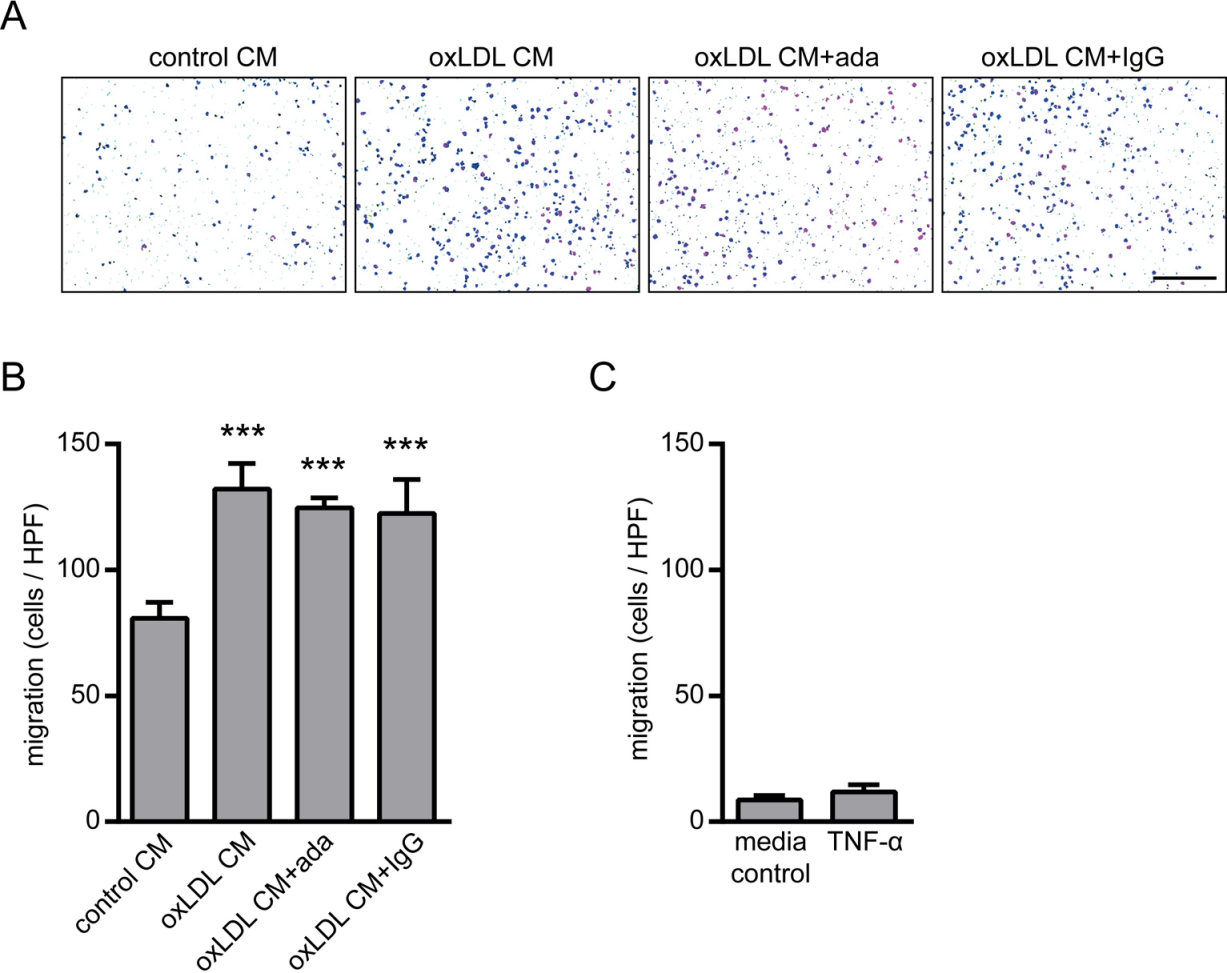
Effect of TNF-α inhibition with adalimumab on migration of THP-1 monocytes. (A) Images depicting crystal violet stained THP-1 cells which migrated across the membrane of transwell inserts (pore size 8 μm) to the lower side after incubation with conditioned media from oxLDL stimulated THP-1 macrophages (oxLDL CM) with or without adalimumab (ada) for 4 hours. Representative pictures are shown. (B) Migrated cells per high power field were quantified. (C) Crystal violet stained THP-1 monocytes which migrated in response to TNF-α (10 ng/mL) across the membrane of transwell inserts (pore size 8 μm) to the lower side were quantified per high power field. Conditioned medium from unstimulated THP-1 macrophages (control CM), IgG isotype (1 μg/mL) and medium were used as control. Scale bar = 200 μm. Representative pictures are shown. ***P<0.001 vs. control CM. n = 3–4 replicated experiments.

Since TNF-α-induced expression of adhesion molecules is critically involved in the attachment of monocytes to the endothelium during the initial phase of atherosclerotic plaque development [10,24] we next investigated the adhesion of THP-1 monocytes to a confluent monolayer of HUVECs under static conditions as well as under in vivo even more relevant flow conditions. Adhesion of THP-1 monocytes was strongly enhanced when HUVECs were incubated with oxLDL CM which was completely blocked by adalimumab (Fig 4A, 4B, 4D and 4E). As expected, recombinant TNF-α significantly enhanced THP-1 monocyte adhesion (Fig 4C and 4F). Next we investigated migration of THP-1 monocytes using the transwell system. We observed a significant increase in THP-1 monocyte migration in response to oxLDL CM but no inhibitory effect with adalimumab (Fig 5A and 5B). Accordingly, we did not observe migration of THP-1 monocytes using TNF-α as a control (Fig 5C) indicating that maybe other ox-LDL-induced factors from THP-1 macrophages (Fig 1B) were responsible for this effect.

### The TNF-α inhibitor adalimumab inhibits endothelial leakage

**Fig 6:**
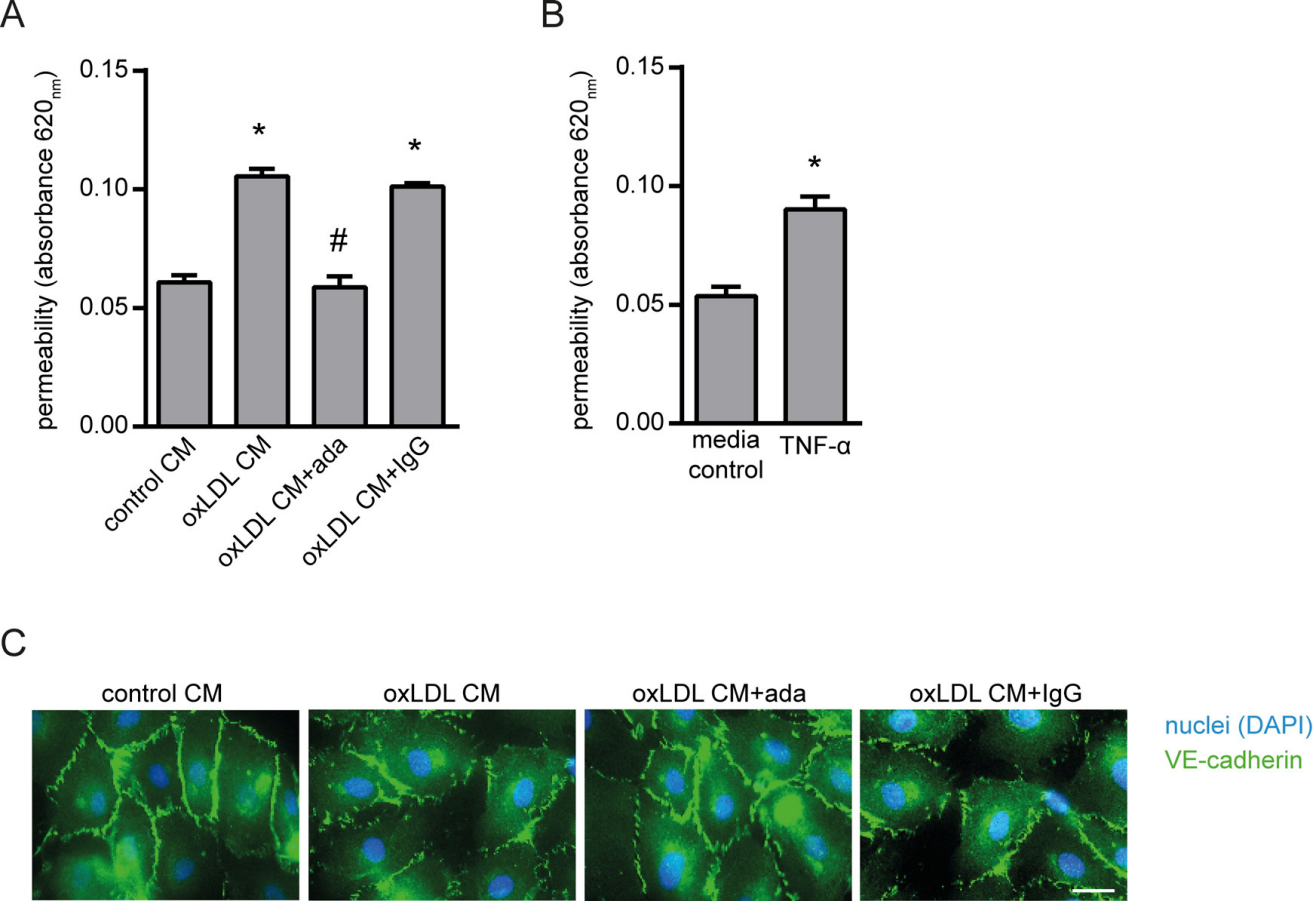
Effect of TNF-α inhibition with adalimumab on endothelial permeability. A confluent monolayer of HUVECs on the membrane of transwell inserts (pore size 0.4 μm) was incubation with (A) conditioned media from oxLDL-stimulated THP-1 macrophages (oxLDL CM) with or without adalimumab (ada) or (B) with TNF-α (10 ng/mL) for 4 hours. Diffusion of Evan´s blue bound BSA across the endothelial monolayer from the luminal to the abluminal chamber was quantified by measuring absorbance at 620 nm of the abluminal chamber after additional 3 hours. (C) VE-cadherin immunofluorescence staining visualizes endothelial cell-to-cell adherens junctions. Conditioned medium from unstimulated THP-1 macrophages (control CM), IgG isotype (1 μg/mL) and medium were used as control. Scale bar = 25 μm. Representative pictures are shown. *P<0.05 vs. control CM or medium, ^#^P<0.05 vs. oxLDL CM, n = 3–4 replicated experiments.

Besides adhesion molecule expression, increased endothelial permeability with increased leucocyte invasion and augmented lipid uptake into the diseased vessel wall is another well-known process in early atherosclerosis and likewise promoted by TNF-α [25,26]. Using the oxLDL CM, we next investigated its effect on endothelial permeability again taking advantage of the transwell system. As observed, oxLDL CM enhanced the vascular permeability of a confluent endothelial monolayer which resulted in increased diffusion of Evan’s blue bound BSA from the luminal to the abluminal chamber. Inhibition of TNF-α by adalimumab significantly inhibited Evan´s blue perfusion (Fig 6A). Consequently, we observed enhanced endothelial permeability with TNF-α (Fig 6B). In addition, we visualized endothelial cell-to-cell adherens junctions by vascular endothelial (VE)-cadherin staining. Immunofluorescence shows loss of endothelial cell integrity with partial gap formation of the monolayer in response to oxLDL CM which was prevented by adalimumab (Fig 6C).

### Enriched vascular deposition of adalimumab

**Fig 7:**
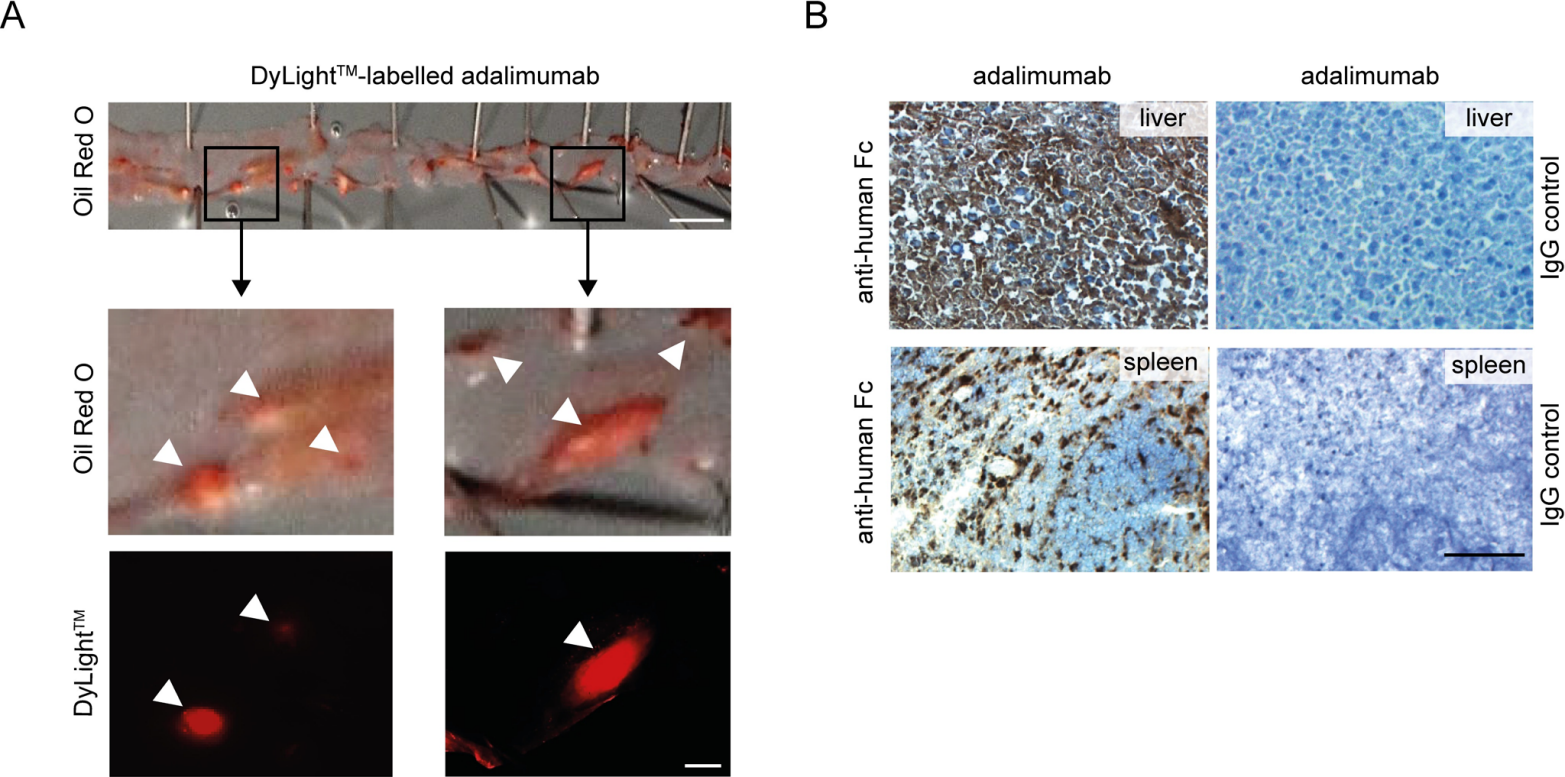
Deposition of adalimumab in aorta, liver and spleen. (A) Aortas of hypercholesterolemic Ldlr^‒/‒^ mice were washed and prepared en face 12 hours after injection of DyLight-549-labelled adalimumab (8.0 mg/kg, i.p.). Fluorescence images (bottom panels) from the aorta were captured before Oil Red O staining (top panels). Lesion area and vascular fluorescence accumulation are indicated by white arrows. Scale bars = 2.5 mm (top panel) and 200 μm (bottom panels). One representative experiment is shown. (B) Immunohistochemical analysis of adalimumab deposition with anti-human Fc or IgG control antibodies in liver and spleen of hypercholesterolemic Ldlr^‒/‒^ mice 12 hours after injection of adalimumab (8.0 mg/kg, i.p.). Scale bar = 100 μm. Representative pictures are shown.

Finally, we studied particularly the vascular deposition of adalimumab in hypercholesterolemic mice. For that purpose, we analyzed en face preparations of aortas 12 hours after injection of DyLight^TM^ 549-labelled adalimumab into Ldlr^‒/‒^ mice which were on a high fat, high cholesterol diet for 6 weeks which reflected early atherosclerosis. Of note, we observed enriched fluorescent-labelled adalimumab mainly in major Oil red O-positive lesions documenting good tissue penetration of the drug into the atherosclerotic plaque (Fig 7A), without background fluorescence of the vessel wall after vehicle control injection into hypercholesterolemic Ldlr^‒/‒^ mice (S3A Fig). Using an antibody specific for the human Fc fragment of adalimumab, we also observed the accumulation of adalimumab in organs such as liver and spleen (Fig 7B and S3B Fig).

## Discussion

We here report that the TNF-α antagonist adalimumab effectively blocks effects of TNF-α released from oxLDL-stimulated THP-1 macrophages on endothelial cells, i.e. increased endothelial adhesion molecule expression, subsequent monocyte adhesion and endothelial leakage (S4 Fig). These processes are known as early events in atherosclerotic plaque development which potentially renders adalimumab eligible for the treatment of vascular inflammation.

Hyperlipidemia is known as a crucial trigger for atherosclerotic vascular disease. Augmented cholesterol deposition in the vascular wall and particularly subsequent oxLDL accumulation is an early key event of atherosclerotic plaque formation [1,2]. In this regard, oxLDL promotes directly the recruitment and retention of monocytes into the vessel wall which consecutively differentiate into tissue macrophages. However, oxLDL also induces the secretion of high amounts of pro-inflammatory cytokines and chemokines from these cells which further amplifies vascular inflammation and plaque progression [27]. TNF-α is one of such cytokines which is released from macrophages upon oxLDL incubation [23] and fires many pro-inflammatory processes in early atherosclerosis [3]. The crucial role of TNF-α in atherosclerosis is well supported by scientific evidence. Disruption of the TNF-α gene locus in different experimental mouse models consistently revealed diminished lesion progression [16–18] and demonstrated that TNF-α is actively involved in the progression of atherosclerosis. However, little is known if pharmacological TNF-α inhibitors could be successfully used to limit progression of atherosclerosis in experimental models. To our best knowledge, so far just two studies exist using such an approach. Brånén et al. treated Apoe^‒/‒^ mice with recombinant soluble TNF receptor I and observed 75% reduction in lesion size [16]. In addition, Tuleta and co-workers found improved endothelial function and reduced atherosclerotic plaques in Apoe^‒/‒^ mice subjected to chronic intermitted hypoxia and treated with the TNF-α inhibitor infliximab [28].

In this regard, TNF-α has been found to play not only a pivotal role in the pathogenesis of atherosclerosis [3] but also in many other chronic inflammatory diseases, e.g. asthma, chronic obstructive pulmonary disease, rheumatoid arthritis and inflammatory bowel disease [4–7]. Thus, it is not surprising that TNF-α agonist have already entered clinic routine a while ago. These pharmacological agents include etanercept, a soluble TNF-α receptor antagonist and antibodies targeting TNF-α, i.e. adalimumab and golimumab a fully human monoclonal antibody, infliximab, a chimeric monoclonal antibody and certolizumab a humanized F_ab_ fragment linked to polyethylene glycol [8,29]. These drugs have in common that they efficiently block the biological effects of TNF-α but show different clinical efficiency which might be explained by different mode of action and pharmacokinetics. In the present study we used adalimumab which was the first complete human monoclonal antibody and has the widest range of indications among all TNF-α inhibitors. Adalimumab is approved for the treatment of inflammatory diseases such as rheumatoid arthritis, psoriatic arthritis, plaque psoriasis, inflammatory bowel diseases (Crohn's disease, ulcerative colitis, pediatric Crohn's disease, and intestinal Behçet's disease), ankylosing spondylitis, axial spondyloarthritis and juvenile idiopathic arthritis [30].

Of all these inflammatory diseases rheumatoid arthritis share the highest similarity to atherosclerosis [9], but a large-scale clinical trial addressing a potential benefit of adalimumab for patients with coronary artery disease or peripheral artery disease has not yet been carried out. In other words, little is known so far about the effect of adalimumab on atherosclerosis. Of note, patients with rheumatoid arthritis have an increased prevalence of atherosclerosis [31]. However, with rheumatoid arthritis patients on adalimumab therapy, there are several smaller studies existing showing promising results on study endpoints related to early atherosclerotic vascular disease. For example, adalimumab therapy is associated with improvement of endothelial function, arrest of intima-media wall thickening and decreased C-reactive protein levels [32–34]. To date, the underlying mechanism of TNF-α inhibition by adalimumab on sub-clinic atherosclerosis is not fully comprehended. In this regard, a number of molecular and cellular processes of early atherosclerotic events are known to be crucially promoted by TNF-α. These include up-regulation of adhesion molecules [10–12] subsequent monocyte adhesion [10,24] and increased endothelial permeability resulting in disruption of endothelial barrier function [25,26]. In our present study, we used human cell culture models and show that all these processes could by suppressed by adalimumab. Finally, we took advantage of an experimental murine model of early atherosclerosis and observed enriched fluorescent-labelled adalimumab mainly in atherosclerotic plaques which documents good penetration of the drug into the tissue of our interest. Our results demonstrate that adalimumab is capable of blocking TNF-α –which is released in high amounts from plaque macrophages in response to oxLDL–and its downstream pro-atherosclerotic effects on endothelial cells and monocytes.

## Conclusion

Since adalimumab is an exclusive human specific TNF-α inhibitor we could not test its effect on atherosclerotic plaque development in an experimental mouse model of atherosclerosis. In our in vitro studies, we have identified effects of adalimumab to prevent fundamental inflammatory processes such as up-regulation of endothelial adhesion molecules, subsequent monocyte adhesion and endothelial leakage, suggesting a potential inhibitory influence of adalimumab on atherosclerotic plaque development. Accordingly, adalimumab–which is widely used for the therapy of patients with rheumatoid arthritis–likewise limits vascular inflammation which is of particular interest because these patients have an increased risk for cardiovascular disease.

## Supporting Information

S1 Fig Characterization of THP-1 cells. (A) Flow chart of the experimental setting. (B) Maturation of THP-1 macrophages. THP-1 monocytes were treated with PMA for 48 hours and analyzed for THP-1 macrophage maturation markers. Cell surface expression of CD11b and CD11c was verified by flow cytometry using APC- and PerCP/Cy5.5-labelled antibodies (filled red and blue graph), respectively. Vehicle-treated THP-1 monocytes (filled grey graph) and appropriate labelled isotype IgG (dashed open graph) were used as control. Representative pictures are shown. (C) Scavenger receptor expression. mRNA expression of CD36 and LOX1 in THP-1 monocytes (Mon) and in THP-1 macrophages (Mac) after treatment with PMA for 48 hours was determined by real time PCR. **P<0.01, ***P<0.001 vs. THP-1 Mon, n = 4 replicated experiments. Foam cell formation of THP-1 macrophages is demonstrated by the uptake of Dil-labelled oxLDL for 4 hours by (D) flow cytometry and (E) fluorescence microscopy. nLDL (10 μg/mL) was used as control. nLDL (filled orange graph), oxLDL (filled green graph), untreated (dashed open graph). Scale bar = 50 μm. Representative pictures are shown.
(TIF)

S2 Fig Cell cytotoxicity measurements. Analysis of cell cytotoxicity of adalimumab on endothelial cells following incubation for 12 hours at the indicated concentrations. Cells were incubated for additional 6 hours in the presence of 10% alamar blue. Absorbance of oxidized alamar blue was measured at 600 nm. Cells under continuous EGM-2 conditions, IgG isotype control (10 μg/mL) and treated with 0.1% Triton-X served as control. *P<0.05 vs. control. One representative experiment is shown.
(TIF)

S3 Fig Controls of adalimumab deposition in aorta, liver and spleen. (A) Aortas of hypercholesterolemic Ldlr^‒/‒^ mice were washed and prepared en face 12 hours after injection of vehicle control (i.p.). Fluorescence images (bottom panels) from the aorta were captured before Oil Red O staining (top panels). Scale bars = 2.5 mm (top panel) and 200 μm (bottom panels). (B) Immunohistochemical analysis with anti-human Fc or IgG control antibodies in liver and spleen of hypercholesterolemic Ldlr^‒/‒^ mice 12 hours after injection of vehicle control (i.p.). Scale bar = 100 μm. Representative pictures are shown.
(TIF)

S4 Fig Schematic model on the vascular mode of action of adalimumab on pro-atherosclerotic effects of TNF-α. (TIF)
