# Supplementary figures and images for: Targeting Tumor Necrosis Factor-α with Adalimumab: Effects on Endothelial Activation and Monocyte Adhesion
Source: PLoS One. 2016 Jul 28;11(7):e0160145. doi: 10.1371/journal.pone.0160145 (PMC4965117; doi:10.1371/journal.pone.0160145)

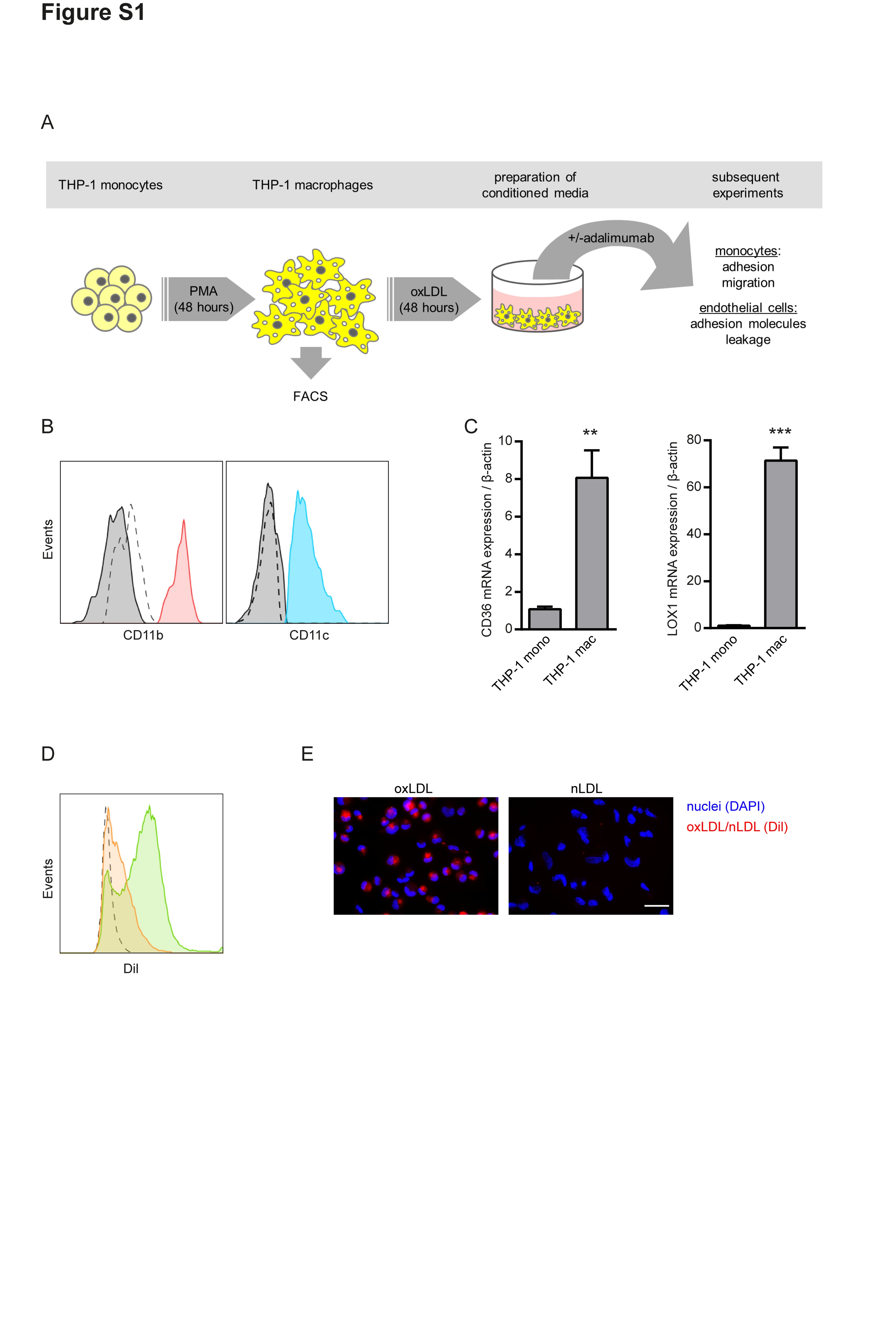

Supplement: S1 Fig — (A) Flow chart of the experimental setting. (B) Maturation of THP-1 macrophages. THP-1 monocytes were treated with PMA for 48 hours and analyzed for THP-1 macrophage maturation markers. Cell surface expression of CD11b and CD11c was verified by flow cytometry using APC- and PerCP/Cy5.5-labelled antibodies (filled red and blue graph), respectively. Vehicle-treated THP-1 monocytes (filled grey graph) and appropriate labelled isotype IgG (dashed open graph) were used as control. Representative pictures are shown. (C) Scavenger receptor expression. mRNA expression of CD36 and LOX1 in THP-1 monocytes (Mon) and in THP-1 macrophages (Mac) after treatment with PMA for 48 hours was determined by real time PCR. **P<0.01, ***P<0.001 vs. THP-1 Mon, n = 4 replicated experiments. Foam cell formation of THP-1 macrophages is demonstrated by the uptake of Dil-labelled oxLDL for 4 hours by (D) flow cytometry and (E) fluorescence microscopy. nLDL (10 μg/mL) was used as control. nLDL (filled orange graph), oxLDL (filled green graph), untreated (dashed open graph). Scale bar = 50 μm. Representative pictures are shown. (TIF) [file pone.0160145.s001.tif]

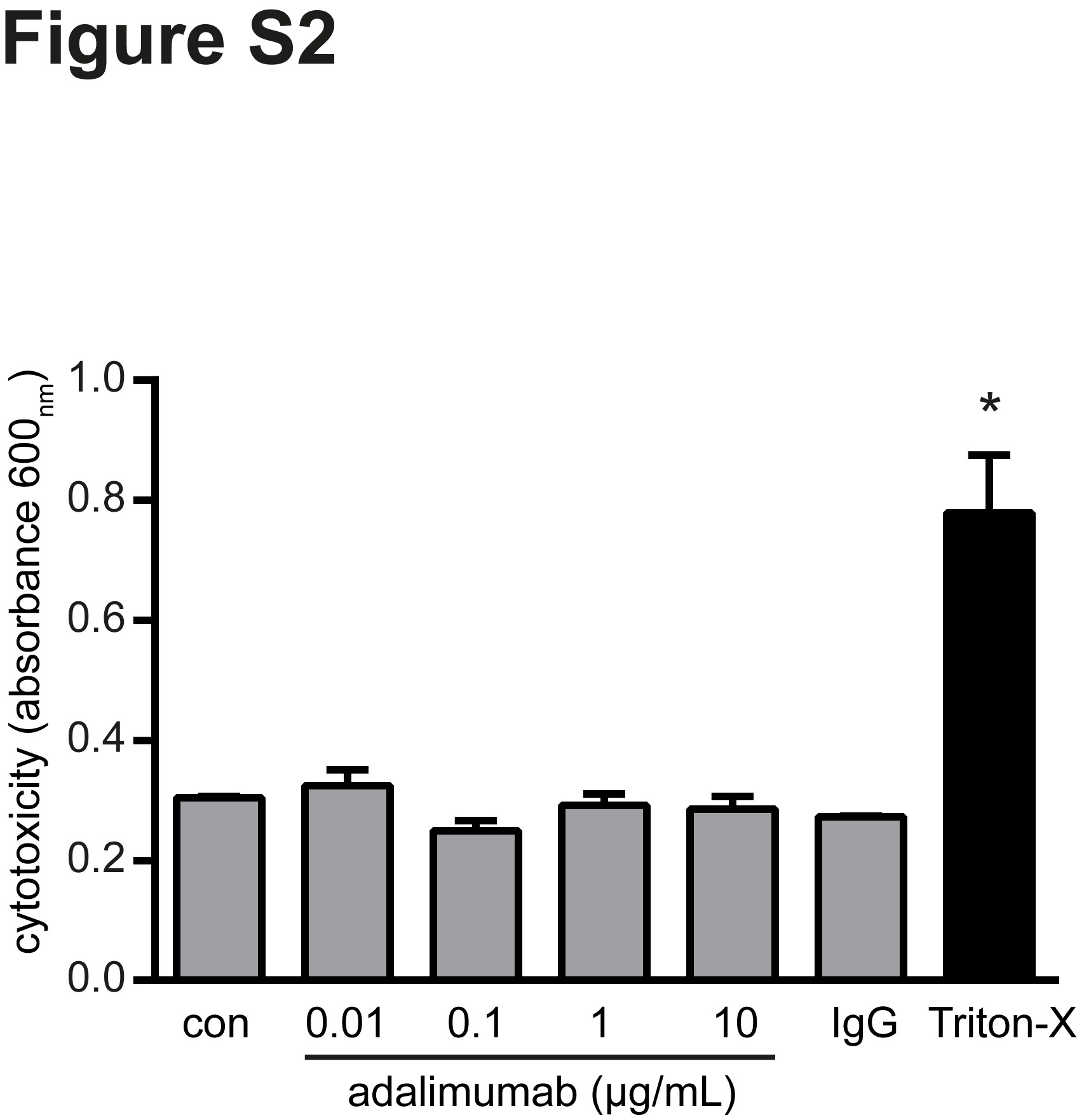

Supplement: S2 Fig — Analysis of cell cytotoxicity of adalimumab on endothelial cells following incubation for 12 hours at the indicated concentrations. Cells were incubated for additional 6 hours in the presence of 10% alamar blue. Absorbance of oxidized alamar blue was measured at 600 nm. Cells under continuous EGM-2 conditions, IgG isotype control (10 μg/mL) and treated with 0.1% Triton-X served as control. *P<0.05 vs. control. One representative experiment is shown. (TIF) [file pone.0160145.s002.tif]

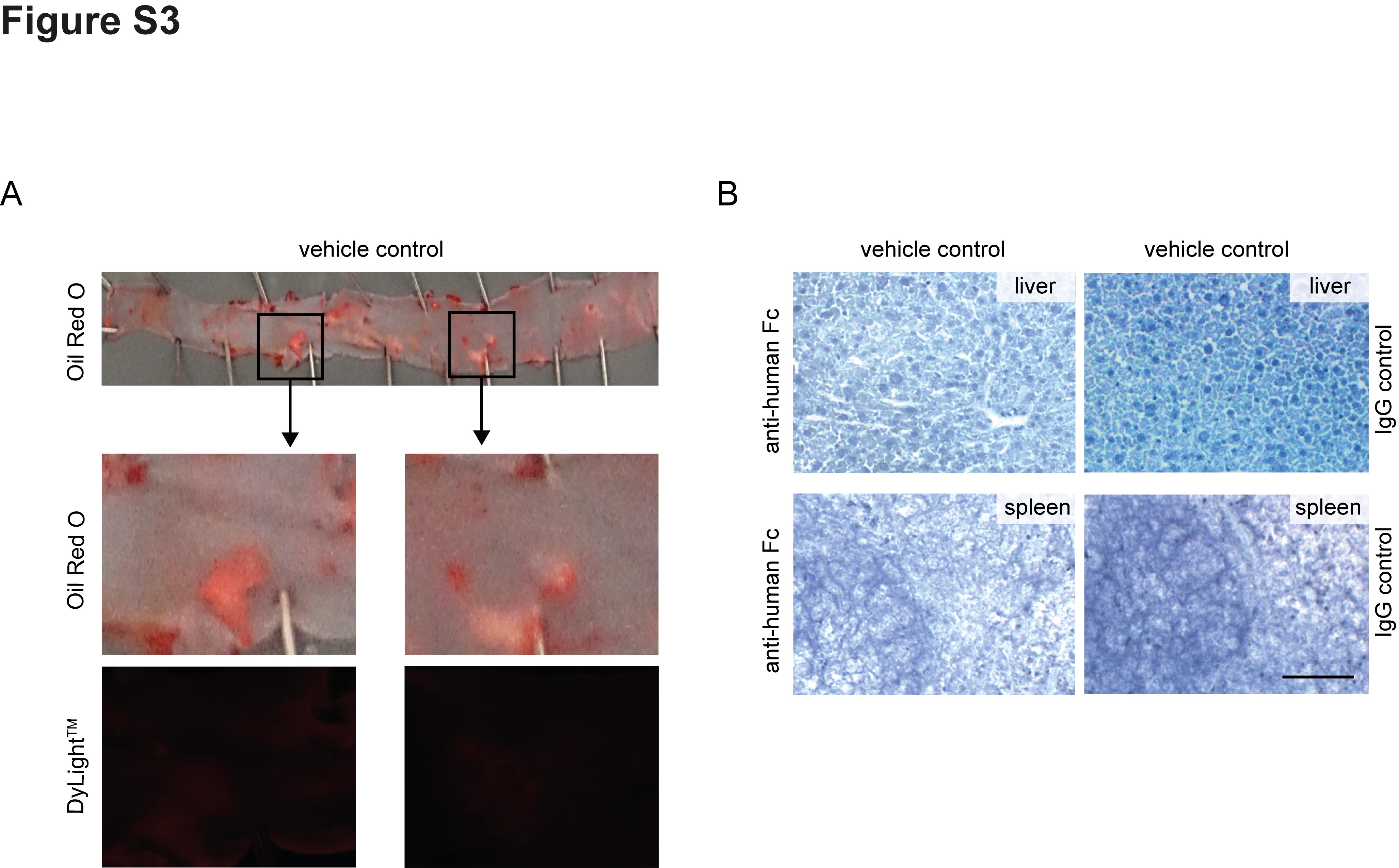

Supplement: S3 Fig — (A) Aortas of hypercholesterolemic Ldlr‒/‒ mice were washed and prepared en face 12 hours after injection of vehicle control (i.p.). Fluorescence images (bottom panels) from the aorta were captured before Oil Red O staining (top panels). Scale bars = 2.5 mm (top panel) and 200 μm (bottom panels). (B) Immunohistochemical analysis with anti-human Fc or IgG control antibodies in liver and spleen of hypercholesterolemic Ldlr‒/‒ mice 12 hours after injection of vehicle control (i.p.). Scale bar = 100 μm. Representative pictures are shown. (TIF) [file pone.0160145.s003.tif]

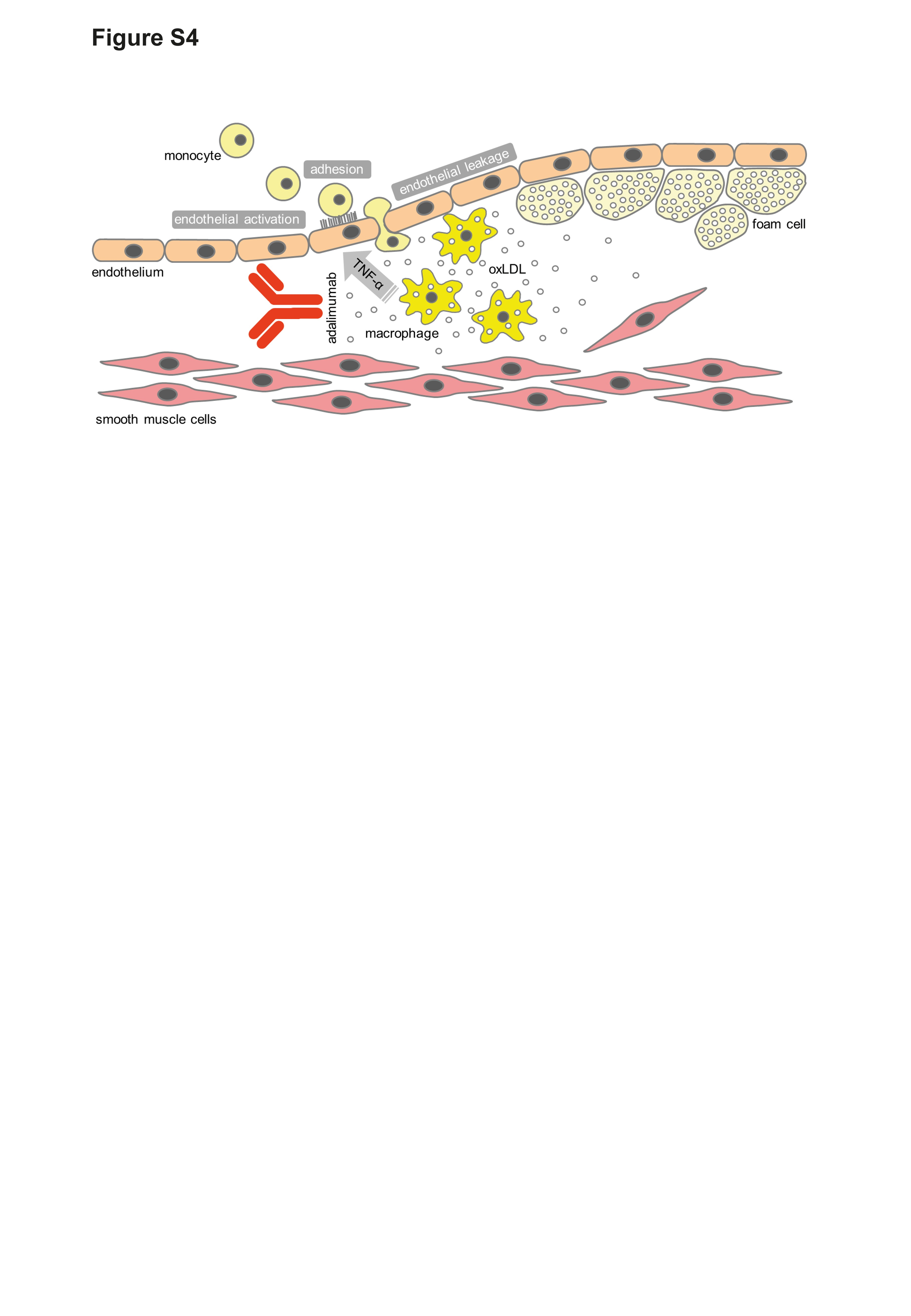

Supplement: S4 Fig — (TIF) [file pone.0160145.s004.tif]
